# *Pasteurella Multocida* Toxin Prevents Osteoblast Differentiation by Transactivation of the MAP-Kinase Cascade via the Gα_q/11_ - p63RhoGEF - RhoA Axis

**DOI:** 10.1371/journal.ppat.1003385

**Published:** 2013-05-16

**Authors:** Peter Siegert, Gudula Schmidt, Panagiotis Papatheodorou, Thomas Wieland, Klaus Aktories, Joachim H. C. Orth

**Affiliations:** 1 Institut für Experimentelle und Klinische Pharmakologie und Toxikologie, Albert-Ludwigs-Universität Freiburg, Freiburg, Germany; 2 Hermann–Staudinger–Graduiertenschule Universität Freiburg, Freiburg, Germany; 3 Institute of Experimental and Clinical Pharmacology and Toxicology, Medical Faculty Mannheim, University of Heidelberg, Mannheim, Germany; 4 BIOSS Centre for Biological Signalling Studies, Universität Freiburg, Freiburg, Germany; Northwestern University, Feinberg School of Medicine, United States of America

## Abstract

The 146-kDa *Pasteurella multocida* toxin (PMT) is the main virulence factor to induce *P. multocida*-associated progressive atrophic rhinitis in various animals. PMT leads to a destruction of nasal turbinate bones implicating an effect of the toxin on osteoblasts and/or osteoclasts. The toxin induces constitutive activation of Gα proteins of the G_q/11_-, G_12/13_- and G_i_-family by deamidating an essential glutamine residue. To study the PMT effect on bone cells, we used primary osteoblasts derived from rat calvariae and stromal ST-2 cells as differentiation model. As marker of functional osteoblasts the expression and activity of alkaline phosphatase, formation of mineralization nodules or expression of specific transcription factors as osterix was determined. Here, we show that the toxin inhibits differentiation and/or function of osteoblasts by activation of Gα_q/11_. Subsequently, Gα_q/11_ activates RhoA via p63RhoGEF, which specifically interacts with Gα_q/11_ but not with other G proteins like Gα_12/13_ and Gα_i_. Activated RhoA transactivates the mitogen-activated protein (MAP) kinase cascade via Rho kinase, involving Ras, MEK and ERK, resulting in inhibition of osteoblast differentiation. PMT-induced inhibition of differentiation was selective for the osteoblast lineage as adipocyte-like differentiation of ST-2 cells was not hampered. The present work provides novel insights, how the bacterial toxin PMT can control osteoblastic development by activating heterotrimeric G proteins of the Gα_q/11_-family and is a molecular pathogenetic basis for understanding the role of the toxin in bone loss during progressive atrophic rhinitis induced by *Pasteurella multocida*.

## Author Summary

*Pasteurella multocida* causes as a facultative pathogen various diseases in men and animals. One induced syndrome is atrophic rhinitis, which is a form of osteopenia, mainly characterized by facial distortion due to degradation of nasal turbinate bones. Strains, which especially affect bone tissue, produce the protein toxin *P. multocida* toxin (PMT). Importantly, PMT alone is capable to induce all symptoms of atrophic rhinitis. To cause osteopenia PMT influences the development and/or activity of specialized bone cells like osteoblasts and osteoclasts. Recently, we could identify the molecular mechanism of PMT. The toxin constitutively activates certain heterotrimeric G proteins by deamidation. Here, we studied the effect of PMT on the differentiation of osteoblasts. We demonstrate the direct action of PMT on osteoblasts and osteoblast-like cells and as a consequence inhibition of osteoblastic differentiation. Moreover, we revealed the underlying signal transduction pathway to impair proper osteoblast development. We show that PMT activates small GTPases in a Gα_q/11_ dependent manner via a non-ubiquitously expressed RhoGEF. In turn the mitogen-activated protein kinase pathway is transactivated leading to inhibition of osteoblastogenesis. Our findings present a mechanism how PMT hijacks host cell signaling pathways to hinder osteoblast development, which contributes to the syndrome of atrophic rhinitis.

## Introduction

Bone tissue is a common target for bacterial infections. Diseases like caries, periodontitis or osteomyelitis are due to infections by *Streptococcus mutans*, *Actinobacillus actinomycetemcomitans* or *Staphylococcus aureus* inter alia. The mechanism of bacterial-induced bone damage may be caused by factors released from pathogens, which interact with bone matrix or affect bone cells, or by bacteria which directly invade bone cells to initiate pathological changes [1]. One of the skeleton affecting bacteria is *Pasteurella multocida*, which causes various diseases in men and an`imals. As a commensal *P. multocida* is found mainly in the nasal/pharyngeal space of domesticated and wild animals and is frequently isolated from cat and dog bites [2]. *P. multocida* is directly or as a supportive factor connected to several diseases like haemorrhagic septicaemia in hoofed animals, avian cholera or snuffles in rabbits [3]. In the case of the economically important progressive atrophic rhinitis *P. multocida* has a central role [4]. Atrophic rhinitis is characterized by drastic degeneration of nasal turbinate bones, leading to a shortening and/or twisting of the snout accompanied by growth retardation of young pigs. Besides domesticated pigs, rabbits, wild pigs and cattle show atrophic rhinitis-like symptoms [3].

The causative agent of atrophic rhinitis is *Pasteurella multocida* toxin (PMT), which is produced by capsular type D and some type A strains [5]. Inoculation of PMT alone is sufficient to generate all symptoms of atrophic rhinitis in animals [6]. Bone tissue is constantly rebuilt by the action of osteoblasts and osteoclasts [7]. Accordingly, analysis of nasal turbinates in atrophic rhinitis disclosed effects of PMT on both types of cells. Besides bone resorption areas, a depletion of osteoblasts was reported [8]. In *in vitro* models the toxin inhibits osteoblastic differentiation and stimulates the differentiation of osteoclasts [9]–[11]. Moreover, a PMT-induced activation of RhoA seems to be important for the blockade of osteoblast differentiation [12]. Notably, PMT induces bone destruction but exhibits no obvious cytotoxicity [3], [13].

Up to date a detailed analysis of PMT-activated signaling pathways in osteoblasts was hampered by the fact that the intracellular substrate of the toxin was unknown. Recently, we identified the molecular mechanism of PMT. The toxin stimulates heterotrimeric G protein signaling. In the switch II region of the α-subunit of heterotrimeric G proteins, PMT deamidates a specific Gln residue, which is involved in GTP hydrolysis [14], [15]. Once the α-subunits are deamidated, they have a constitutive active phenotype. PMT targets α-subunits of the Gα_q/11_-, Gα_12/13_- and Gα_i_-family [16]–[20]. A consequence is the activation of multiple signal transduction pathways, leading in a cell type specific manner to strong mitogenicity, anti-apoptotic effects or restructuring of the cytoskeleton [21], [22].

Differentiation and activity of osteoblasts and osteoclasts are tightly regulated. Osteoblast differentiation is stimulated by various factors like BMP, PTH or growth factors as IGF or TGF, acting on different types of receptors [23]. In addition, previous studies showed that various heterotrimeric G proteins and G protein-coupled receptors are involved in the regulation of osteoblast differentiation. Thereby, Gα_s_ and Gα_i_ signaling appear to control differentiation of bone cells in an opposite manner [24], [25]. The opposing effects of Gα_s_ and Gα_i_ on osteoblasts depend at least partly on the regulation of adenylyl cyclase [26]. Furthermore, it was shown that a constitutive active mutant of Gα_q_ blocked differentiation of osteoblasts. Transgenic mice, expressing this mutant in osteoblast progenitors, developed osteopenia [27].

Also the mitogen-activated protein kinase (MAPK) pathway contributes to bone development. However, the data available are inconsistent, because studies provide evidence for positive as well as for negative effects on bone cell development upon MAPK activation [28]–[31].

Elucidation of the molecular mechanism of PMT and recent progress in the understanding of bone cell development prompted us to analyze the effects of PMT on osteoblasts and osteoblast precursors in more detail. Here we present evidence that PMT controls the differentiation of osteoblasts by constitutive activation of Gα_q/11_, subsequent stimulation of RhoA/Rock pathway via p63RhoGEF and transactivation of MAPK cascade.

## Results

### PMT inhibits osteoblastic differentiation in ST-2 cells

**Figure 1:**
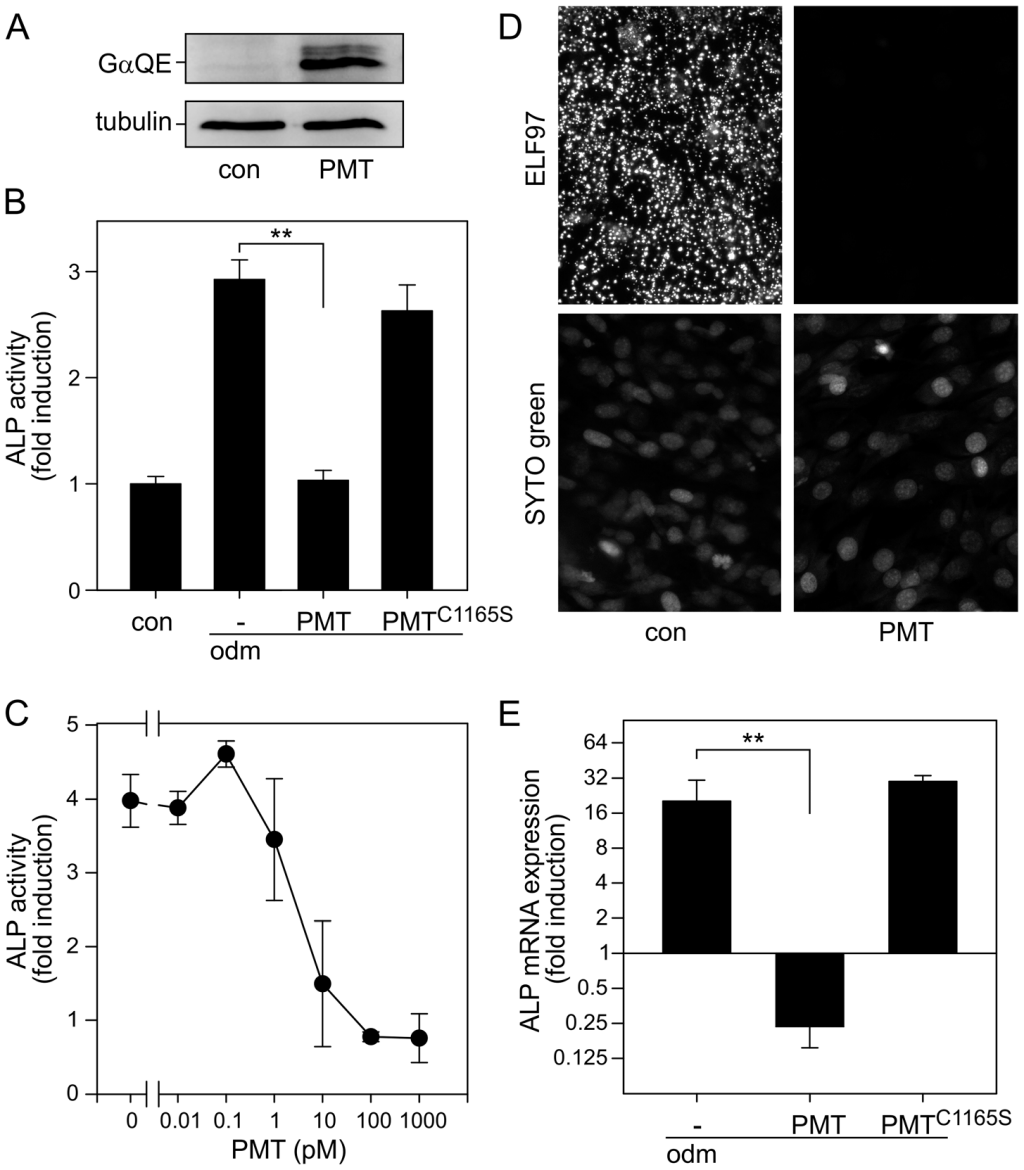
PMT inhibits osteoblast-like differentiation in stromal ST-2 cells. (A) Detection of PMT-deamidated α-subunits of heterotrimeric G proteins. ST-2 cells were treated with or without PMT (1 nM, 20 h). Cells were lysed and subjected to SDS-PAGE followed by immunoblot analysis, utilizing monoclonal rat anti-Gαq Q209E (3G3, anti-QE) and monoclonal mouse anti-tubulin antibody as described in Experimental Procedures. (B) ST-2 cells were incubated with osteoblast differentiation medium (odm) or not (con) in the presence of PMT or PMT^C1165S^ (each 100 pM) for 10 d. Then alkaline phosphatase (ALP) activity was measured. Shown is the fold induction of ALP activity over undifferentiated (con) cells. (C) Concentration-dependent inhibition of ALP activity in odm-treated ST-2 cells by PMT. ST-2 cells were cultured in odm in the presence of PMT at indicated concentrations for 10 d. Then alkaline phosphatase (ALP) activity was measured. Shown is the fold induction of ALP activity over undifferentiated cells. (D) Detection of ALP utilizing enzyme labeled fluorescence ELF97. Odm-treated ST-2 cells were incubated with or without PMT (100 pM) for 10 d and stained with ELF97 for visualization of ALP and SYTO green for nuclei. (E) ST-2 cells were treated as described in A. After 10 d incubation with PMT or PMT^C1165S^ total RNA was extracted, reverse-transcribed, and target sequence was measured by qPCR using mouse ALP-specific primers. Shown are the relative expression values of ALP normalized against a housekeeping gene (S29) and depicted as the ratio of treated (as indicated) and undifferentiated (without odm) samples. Shown are representative experiments out of at least three performed in triplicates and given as mean ± S.E.

To study the effects of PMT on osteoblast differentiation, we used the established cell culture model of ST-2 cells. ST-2 cells are characterized by their potency to differentiate into osteoblast or adipocyte lineages. Thus, in respect to differentiation, the cells are comparable to osteoblast progenitor cells. The primary effect of PMT is the activation of heterotrimeric G proteins by deamidation of a conserved glutamine. Recently, a monoclonal antibody was described, which selectively recognizes the PMT-deamidated G proteins but not the unaffected ones [32]. By using this antibody, we could demonstrate that the toxin-induced deamidation takes place also in ST-2 cells (Fig. 1A). Thus, we studied osteoblastic differentiation by measuring the activity of alkaline phosphatase (ALP) after 10 d, which is an early marker of osteoblasts and indicates proper differentiation. As shown in Figure 1B, PMT but not the catalytically inactive mutant PMT^C1165S^ inhibited the osteoblast formation. Moreover, as determined by the concentration dependent blockade of ALP activity, the toxin was found to be a strong inhibitor of osteoblastic differentiation. 10 pM PMT nearly abolished osteoblastic differentiation (Fig. 1C). Additionally, osteoblastic differentiation can be visualized by using the phosphatase substrate *enzyme labeled fluorescence* (ELF) 97, which specifically stains ALP. Control cells showed strong staining with ELF97, indicating proper osteoblast differentiation, whereas no alkaline phosphatase staining was visible in PMT-treated cells (Fig. 1D). To exclude that PMT induces degradation of ALP, we quantified mRNA levels. In PMT-treated cells ALP expression levels decreased as compared to cells solely cultured in osteoblastic differentiation medium. In contrast, PMT^C1165S^ exhibited no effect on mRNA levels (Fig. 1E).

### PMT does not inhibit adipocyte formation

**Figure 2:**
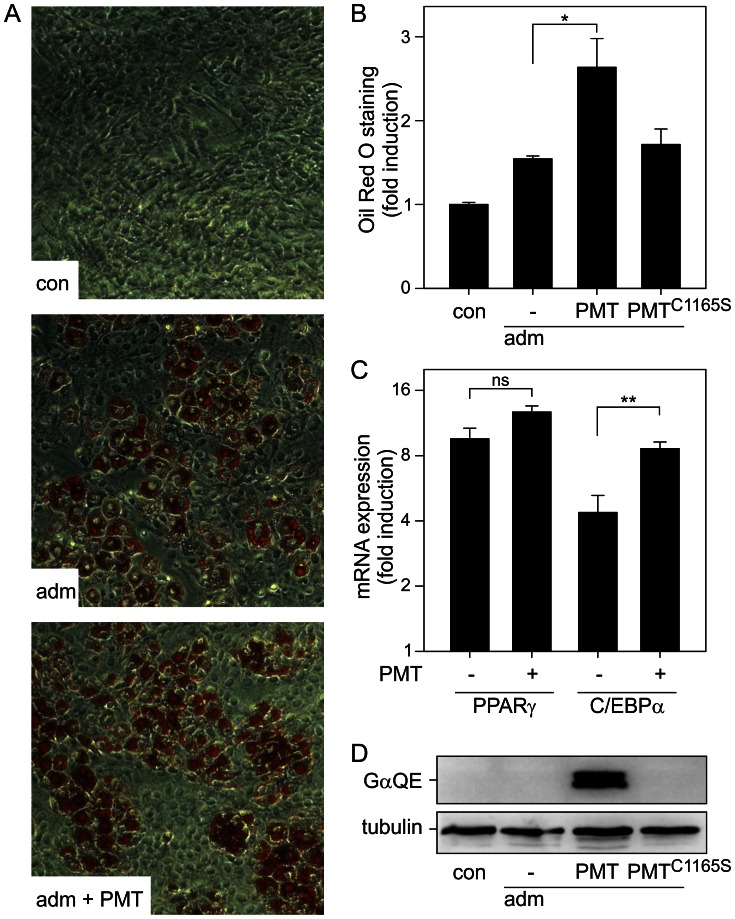
Adipogenesis of stromal ST-2 cells is not affected by PMT. (A) ST-2 cells were incubated with adipocyte differentiation medium (adm) or not (con) in the presence of PMT (100 pM). After 10 d incubation Oil Red O staining was performed to visualize lipid containing vacuoles indicating adipogenesis. (B) Quantification of Oil Red O staining. ST-2 cells were incubated with adm or not (con) in the presence of PMT or PMT^C1165S^ (each 100 pM) for 10 d. After staining cells with Oil Red O, the dye was extracted and quantified. (C) Expression of adipogenic markers is not negatively affected by PMT. ST-2 cells were incubated with or without PMT (100 pM) for 10 d, mRNA was extracted and qPCR was performed, subsequently. Shown are the relative expression values of PPARγ and C/EBPα normalized against housekeeping gene (S29) and depicted as the ratio of treated (as indicated) and undifferentiated (without adm) samples. (D) Detection of PMT-deamidated α-subunits of heterotrimeric G proteins. ST-2 cells were treated as described in A. Cell lysates were subjected to SDS-PAGE followed by immunoblot analysis utilizing monoclonal rat anti-Gαq Q209E (3G3, anti-QE) and monoclonal mouse anti-tubulin antibody. Shown are representative experiments out of at least three performed in triplicates and given as mean ± S.E.

Because ST-2 cells possess the potency to differentiate to diverse lineages, we tested whether PMT also affects differentiation into the adipocyte lineage. Induction of adipocyte differentiation was monitored by the formation of lipid vacuoles. ST-2 cells were incubated for 6 d with adipocyte differentiation medium and lipid droplets were visualized by Oil Red O staining. As shown in Figure 2A, coincubation with PMT did not impair adipocyte differentiation. In contrast, PMT but not PMT^C1165S^, even increased lipid droplet formation compared to the induction by differentiation medium alone (Fig. 2B). The increase in Oil Red O staining could be due to more intense staining or more cells showing staining. To address this question we counted positive- and un-stained cells. PMT treatment significantly increased the total cell number (adipocyte differentiation medium (adm) 183, ±21 SD; adm+PMT 255, ±20 SD, p<0.01, n = 8) and the relative portion of Oil Red O-positive cells (adm 30.5%, ±4% SD; adm+PMT 38.0%, ±3.8% SD, p<0.01, n = 8).

Adipocytes are characterized by the expression of several marker proteins, such as peroxisome proliferator-activated receptor γ (PPARγ) or CCAAT/enhancer-binding family of proteins (C/EBPα). Congruently, quantification of mRNA expression levels of those markers showed no inhibitory effects of PMT (Fig. 2C).

To exclude the possibility that PMT is not able to activate heterotrimeric G proteins under adipogenic conditions, we verified G protein deamidation in an immunoblot. PMT but not PMT^C1165S^ led to deamidation under these conditions (Fig. 2D).

### PMT inhibits functions of primary osteoblasts

**Figure 3:**
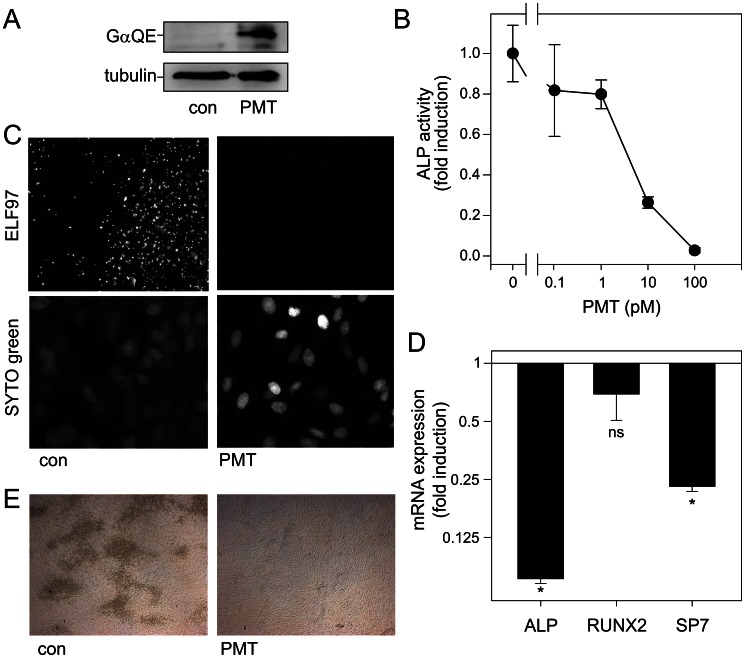
Effect of PMT in primary osteoblasts. (A) Detection of PMT-deamidated α-subunits of heterotrimeric G proteins. Primary osteoblasts were treated with or without PMT (1 nM, 20 h). Cell lysates were subjected to SDS-PAGE followed by immunoblot analysis utilizing monoclonal rat anti-Gαq Q209E (3G3, anti-QE) and monoclonal mouse anti-tubulin antibody as described. (B/C) Influence of PMT on ALP activity and ELF97 staining. Primary osteoblasts derived from rat calvariae were cultivated in odm with indicated concentrations of PMT for 4 d. ALP activity was measured (B) or ALP was stained with ELF97 and syto green for nuclei staining (C). (D) mRNA expression of ALP and bone differentiation regulating transcription factors is shown. odm-treated primary osteoblasts were incubated with or without PMT (1 nM) for 4 d. mRNA levels of ALPL, RUNX2 and SP7 were determined by qPCR. Shown are the relative expression values against housekeeping gene (HP) demonstrated as the ratio of PMT-treated and untreated samples. (E) Detection of mineralization nodules by van Kossa staining was performed without and with PMT (100 pM) in cultures of primary osteoblasts. Shown are representative experiments out of at least three performed in triplicates and given as mean ± S.E.

A system of primary osteoblasts from calvariae of neonatal rats was used to strengthen our studies. Also these primary osteoblasts were directly targeted by PMT as shown by deamidation of heterotrimeric G proteins (Fig. 3A). For functional studies, primary osteoblasts were cultivated for 4–10 d and ALP activity was determined by the ALP activity assay or the ELF97 staining, respectively. As found for the differentiation of ST-2 cells to the osteoblastic lineage, PMT inhibited ALP activity of primary osteoblasts at picomolar concentrations (Fig. 3B). In the PMT-treated primary osteoblasts no ALP staining was detectable with ELF97 (Fig. 3C). In accordance with the diminished ALP activity, also mRNA levels of ALP were reduced by PMT. Specific transcription factors, e.g. runt-related transcription factor 2 (Runx2) or osterix (SP7) regulate osteoblast differentiation. qPCR analysis of SP7 and Runx2 expression revealed that PMT treatment significantly reduced the expression of SP7 but not Runx2 in primary osteoblasts (Fig. 3D). The formation of mineralization nodules is another marker for osteoblasts. Also mineralization nodules, detected by van Kossa staining, were nearly abolished in PMT-treated samples (Fig. 3E).

### RhoA activation is sufficient to impair osteoblast differentiation

The small GTPase RhoA is one of the downstream effectors of PMT-activated heterotrimeric G proteins [17]. Furthermore, it was shown that RhoA is involved in inhibition of osteoblastic differentiation [12]. Therefore, we tested whether the direct activation of RhoA is sufficient to block osteoblastogenesis.

**Figure 4:**
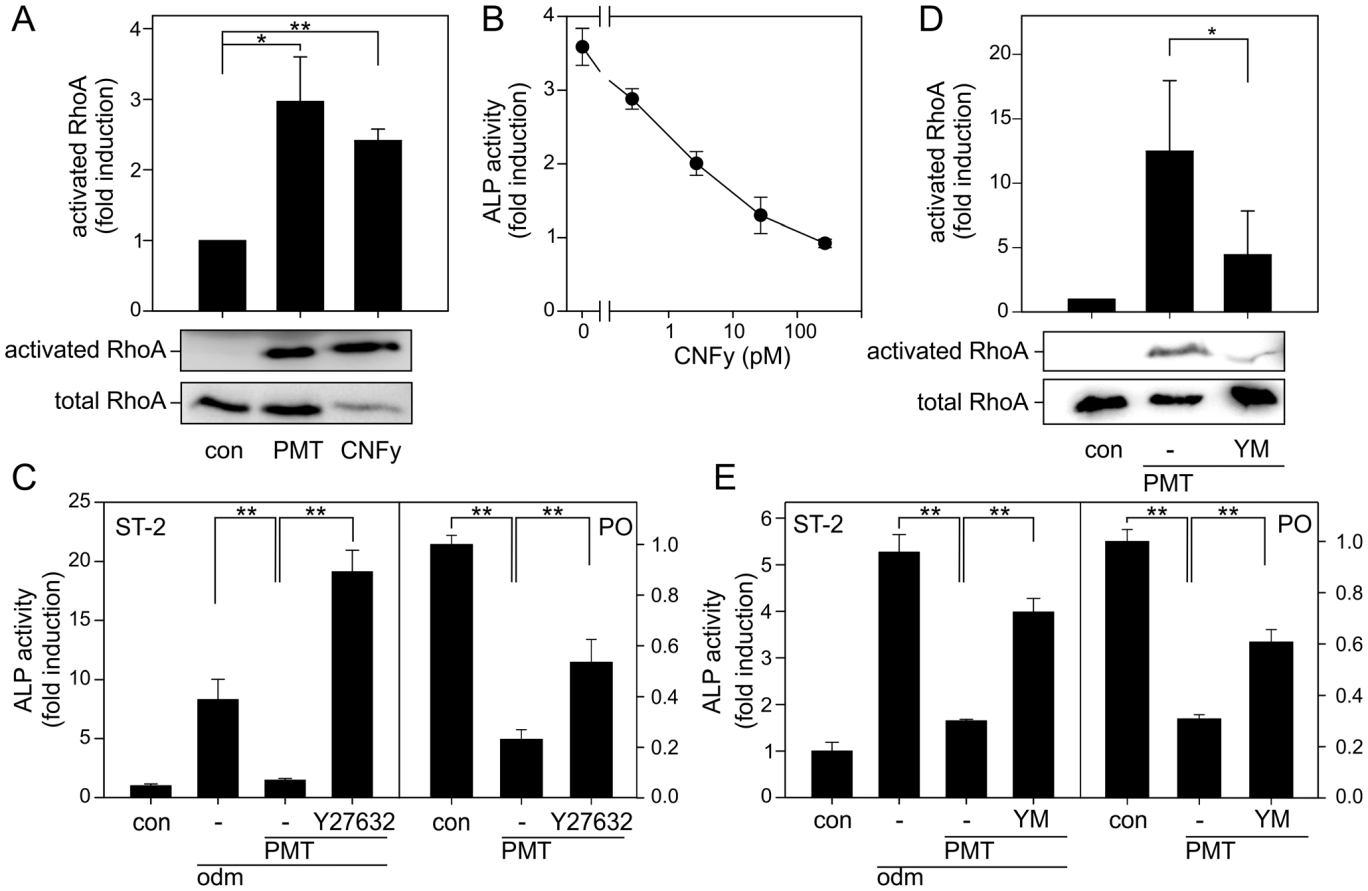
Involvement of Gα_q/11_ and RhoA in PMT-induced inhibition of osteoblastogenesis. (A) RhoA activation by PMT and CNFy in ST-2 cells. Serum-starved ST-2 cells were treated with PMT^wt^ or CNFy (each 1 nM) for 4 h. Thereafter, cells were lysed and pull-down experiments were performed. (B) Influence of CNFy on osteblastogenesis as determined by ALP activity. ST-2 cells were incubated with indicated concentrations of CNFy for 10 d. Then alkaline phosphatase (ALP) activity was measured. Shown is the fold induction of ALP activity over undifferentiated cells. (C) Influence of Rock inhibitor on the inhibitory effect of PMT on osteoblastogenesis in ST-2 cells (left panel) and primary osteoblasts (PO, right panel). ST-2 cells were incubated with odm or not (con) in the presence of PMT (100 pM) and Rock inhibitor Y27632 (1 µM) for 10 d. Primary osteoblasts were incubated with odm in the presence of PMT (1 nM) where indicated and inhibitor for 4 d. Alkaline phosphatase (ALP) activity was measured and demonstrated as fold induction of ALP activity normalized to control cells. (D) Influence of Gα_q/11_ inhibitor YM-254890 on PMT-induced RhoA activation. Serum-starved ST-2 cells were treated with or without YM-254890 (1 µM) 30 min prior incubation with PMT^wt^ (1 nM) for further 4 h. Thereafter, cells were lysed and pull-down experiments were performed. (E) Effects of inhibitor of Gα_q/11_ (YM-254890, YM) on the inhibitory effect of PMT on osteoblastogenesis in ST-2 cells (left panel) and primary osteoblasts (right panel). ST-2 cells were incubated with odm or not (con) in the presence of PMT (100 pM) and Gα_q/11_ inhibitor (YM-254890, 1 µM) for 4 d. Primary osteoblasts were incubated with odm in the presence of PMT (1 nM) where indicated and inhibitor for 4 d. Alkaline phosphatase (ALP) activity was measured and demonstrated as fold induction of ALP activity normalized to control cells. (A/D) Shown are representative immunoblots of at least three performed. Quantification was calculated using MultiGauge and demonstrated as fold induction normalized to untreated cells. Results are given as mean ± S.E. from at least three independent experiments. (B,C,E) Shown are representative experiments out of at least three performed in triplicates and given as mean ± S.E.

To this end, we utilized cytotoxic necrotizing factor (CNF)y from *Yersinia pseudotuberculosis*, which is a specific activator of RhoA [33]. PMT and CNFy activate RhoA as shown by effector pulldown of activated RhoA (Fig. 4A). Treatment of ST-2 cells with increasing concentration of CNFy blocked osteoblastogenesis as measured by the activity of the ALP (Fig. 4B). RhoA activates several effector proteins like the Rho-associated, coiled-coil containing protein kinase (Rock). Pharmacological inhibition of Rock with Y27632 abrogated the inhibitory effect of PMT on osteoblast differentiation (Fig. 4C). In ST-2 cells Y27632 not only reversed the effect of PMT but also increased osteoblastic differentiation. This pro-osteoblastic effect of Y27632 was already previously described [34] and might be due to the reduction of RhoA/Rock signaling below the basal activity level. Our results demonstrate that active RhoA/Rock-signaling inhibits osteoblastogenesis. In turn blockade of RhoA/Rock impairs the PMT effect on differentiation.

The small GTPase RhoA can be stimulated by members of the Gα_q/11_- and Gα_12/13_-family of heterotrimeric G proteins. Via different Rho guanine nucleotide exchange factors (GEF) like p115RhoGEF or p63RhoGEF the heterotrimeric G proteins are linked to RhoA [35], [36]. To determine the Gα_q/11_ dependent portion of PMT-induced RhoA activity, we utilized a specific inhibitor of Gα_q/11_ signaling, YM-254890 [37]. In the presence of YM-254890 the PMT-stimulated RhoA activity was strongly diminished (Fig. 4D). The pivotal role of Gα_q/11_ in PMT-induced RhoA activity prompted us to study the effect of Gα_q/11_ inhibition on osteoblast differentiation. Blockade of PMT-induced Gα_q/11_ activation by YM-254890 abrogated the toxins effect on ALP activity and expression in ST-2 cells (Fig. 4E). Congruently, in primary osteoblasts pharmacological inhibition of RhoA/Rock or Gα_q/11_ prevent the PMT effect on osteoblast differentiation as measured by ALP activity (Fig. 4C/E).

### p63RhoGEF links Gα_q/11_ to RhoA

**Figure 5:**
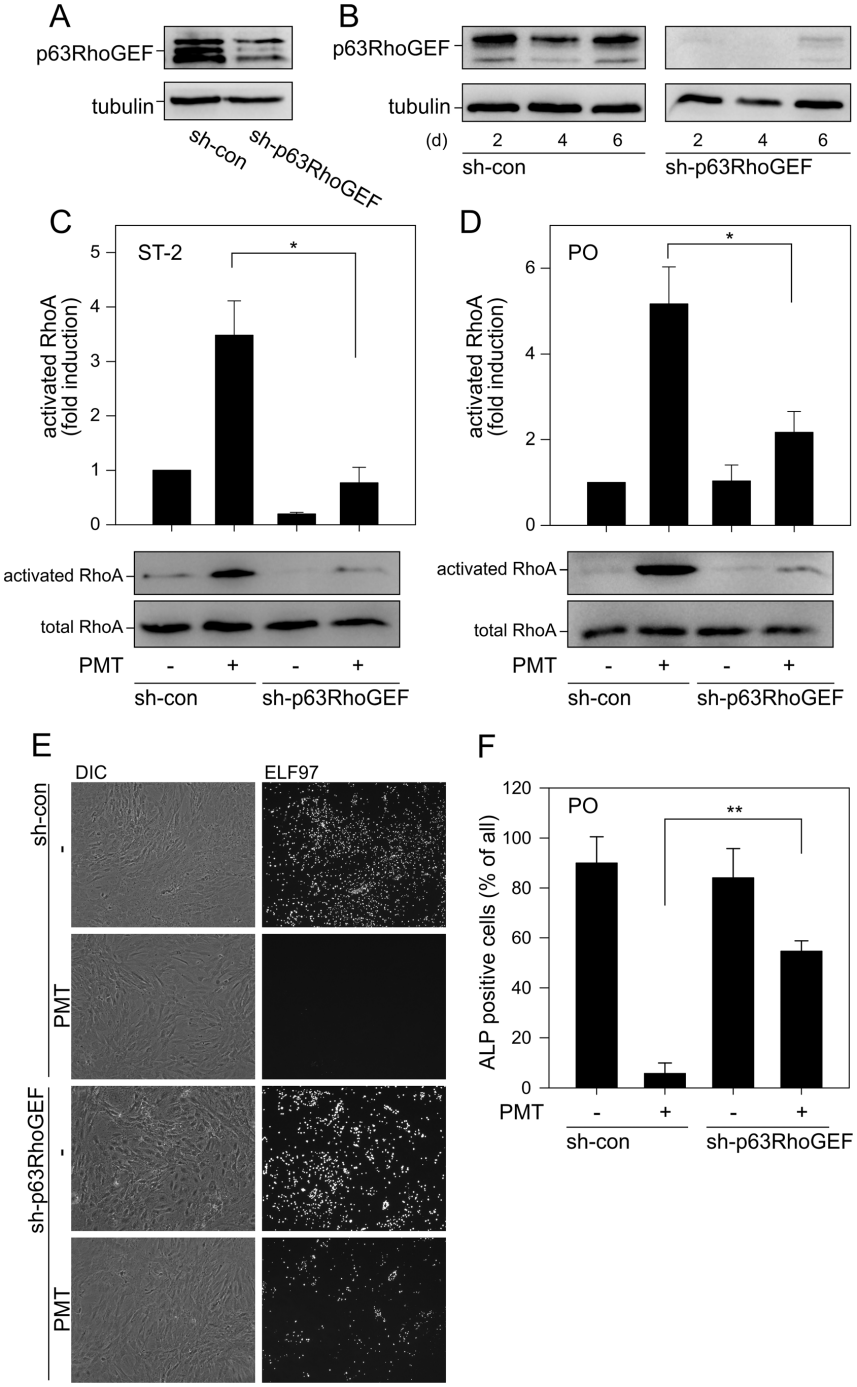
PMT activates RhoA via p63RhoGEF in osteoblastic cells. Figure A/B shows the knockdown of p63RhoGEF. ST-2 cells (A) and primary osteoblasts (B) were transduced with adenoviral vector, encoding for GFP and shRNA for GFP (con) or p63RhoGEF for indicated times. p63RhoGEF expression was analyzed by immunoblot. Equal loading was demonstrated by detection of tubulin. Note: p63RhoGEF is known to have two splice variants and is palmitoylated. All variants are knocked down. (C/D) ST-2 cells (C) and primary osteoblasts (D) were infected with indicated adenovirus for 4 d and PMT-induced RhoA activation was determined by effector pulldown assay. Serum-starved cells were treated with or without PMT^wt^ (1 nM) for 4 h. Thereafter, cells were lysed and pull-down experiments were performed. Shown are representative immunoblot of at least three performed. Quantification was calculated using MultiGauge and demonstrated as fold induction normalized to untreated cells. Results are given as mean ± S.E. from at least three independent experiments. (E) Effects of the knockdown of p63RhoGEF in primary osteoblasts on the inhibitory effect of PMT on osteoblastogenesis. Primary osteoblasts were infected with indicated adenovirus for 4 d. Then PMT was added in odm for further 4 d. ALP activity was determined utilizing the ELF97 assay. Shown are phase contrast micrographs (DIC) of sh-con- and shp63RhoGEF-transduced cells. ELF97 staining is hardly detectable in sh-con-transduced cells after PMT treatment. However, ELF97 staining is detectable in sh-p63RhoGEF-transduced cells even after PMT challenging. (F) Quantification of primary osteoblasts positive stained with ELF97 after treatment as described in D. Results are given as mean ± S.E. from at least three independent experiments.

A specific RhoGEF protein, which couples Gα_q/11_ to RhoA, is p63RhoGEF [36]. This RhoGEF is not ubiquitously expressed [38], however, we detected its expression in ST-2 cells and primary osteoblasts. Next, we addressed the question whether p63RhoGEF is involved in PMT-induced inhibition of osteoblastogenesis. To this end, we depleted endogenous p63RhoGEF by sh-RNA, using an adenoviral transfection system [39]. As shown in Fig. 5 A/B, sh-p63RhoGEF effectively reduced the endogenous content of p63RhoGEF in stromal ST-2 cells and rat primary osteoblasts. Both types of p63RhoGEF-knockdown cells exhibited declined RhoA activity as compared to control cells after PMT treatment (Fig. 5C/D). These results indicated that a predominant portion of PMT-induced RhoA activity depends on the Gα_q/11_-p63RhoGEF axis.

Moreover, we analyzed the ability of PMT to inhibit osteoblast function in primary osteoblasts after p63RhoGEF-knockdown. In control cells PMT inhibited ALP activity as measured by the ELF97 staining. In contrast, in p63RhoGEF-knockdown cells the inhibitory effect of PMT on osteoblastogenesis was significantly abrogated as shown by strong activity of ALP (Fig. 5E/F). By this approach we confirmed that the Gα_q/11_-p63RhoGEF-RhoA axis is of major importance for PMT-dependent inhibition of osteoblast differentiation.

### Transactivation of MAPK-signaling

**Figure 6:**
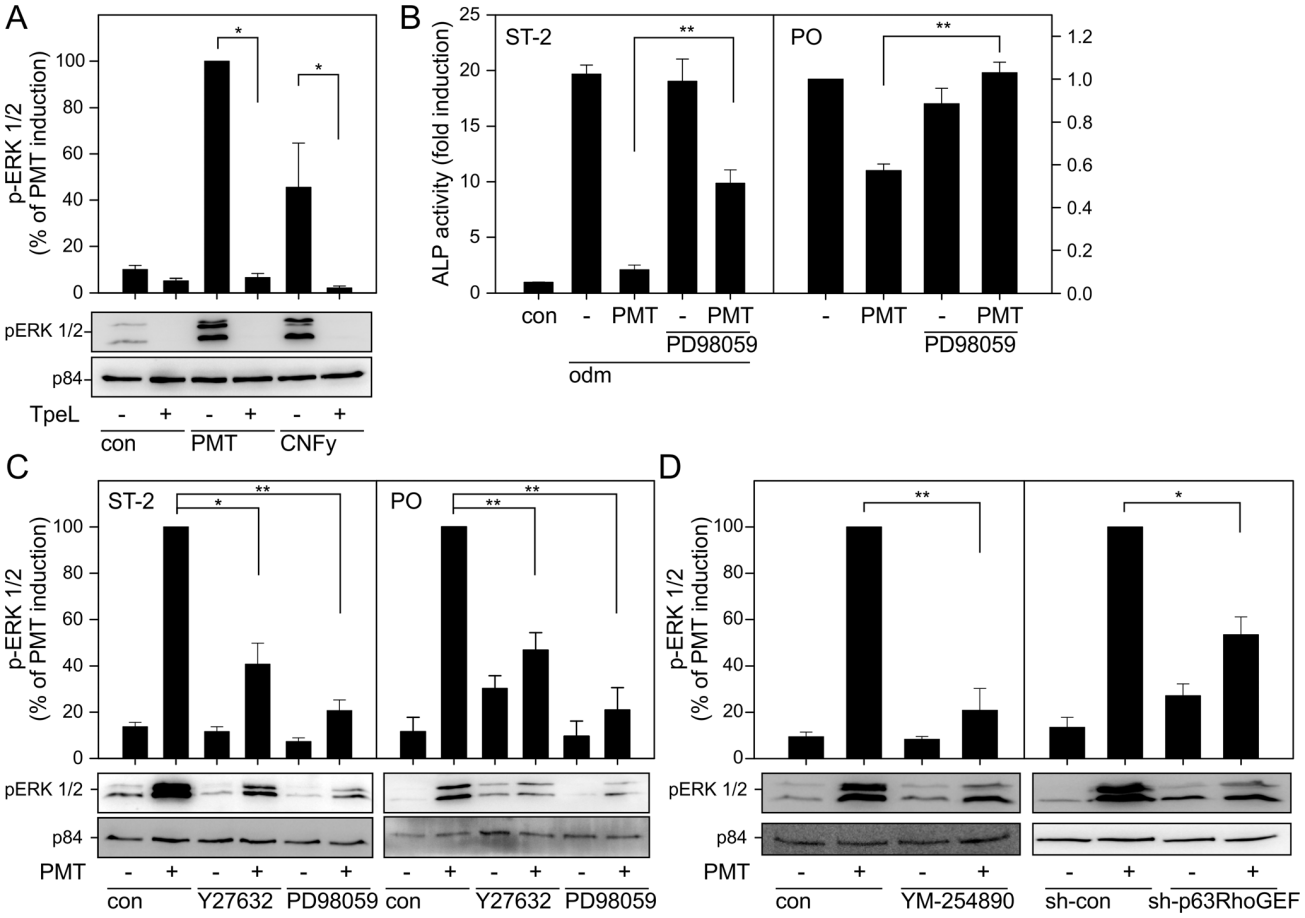
PMT stimulates the mitogen-activated protein kinase signaling pathway via the Gα_q/11_-Rho axis to inhibit osteoblast differentiation. (A) Ras dependency of PMT- and CNFy-induced stimulation of ERK phosphorylation. ST-2 cells were pretreated with Ras inactivating *Clostridium perfringens* toxin TpeL (500 pM, overnight) or not and stimulated with PMT (1 nM) or CNFy (1 nM) for further 5 h. Phosphorylation of ERK was detected as described in Experimental Procedures. (B) Effects of inhibition of MAPK signaling on PMT-induced inhibition of osteoblast differentiation. ST-2 cells were incubated with osteoblast differentiation medium (odm) or not (con) in the presence of PMT (100 pM) and PD98059 (5 µM) where indicated for 4 d (left panel). Primary osteoblasts (PO) were incubated in the presence of PMT (1 nM) and PD98059 (20 µM) where indicated for 4 d (right panel). Then ALP activity was measured and demonstrated as fold induction normalized to control cells. Shown are representative experiments out of at least three performed in triplicates and given as mean ± S.E. (C) Effects of Rho kinase inhibitor Y27632 and MEK-1 inhibitor PD98059 on PMT-induced p-ERK levels. ST-2 cells were pretreated with Y27632 (50 µM) or PD98059 (50 µM) for 30 min. Where indicated an incubation with PMT (1 nM) for 5 h followed. Then phosphorylation of ERK was detected. (D) Effects of inhibition of Gα_q/11_ and knockdown of Gα_q/11_ effector p63RhoGEF on PMT-induced ERK phosphorylation. Cells were pretreated with Gα_q/11_ inhibitor YM-524890 (2.5 µM) for 30 min followed by PMT (1 nM) stimulation for 5 h (left panel). Additionally, ST-2 cells were transduced with adenovirus encoding for sh-p63RhoGEF or sh-con where indicated. Cells were stimulated with PMT (1 nM, 5 h) (right panel) and p-ERK was determined subsequently. Shown are representative immunoblots of at least three performed. Quantification was calculated and demonstrated as fold induction normalized to untreated cells. Results are given as mean ± S.E. from at least three independent experiments.

The mitogen-activated protein kinase (MAPK) pathway has been implicated in regulation of osteogenesis [40]. Therefore, we tested whether PMT stimulates the MAPK pathway in osteoblasts by measuring phosphorylation of the extracellular signal-regulated kinase (ERK) 1/2. To this end serum-starved stromal ST-2 cells were incubated with PMT and ERK activity was determined by an immunoblot approach. PMT strongly enhanced ERK activity as shown in Fig. 6A. Additionally, we studied the effect of the specific RhoA activator CNFy. Similar to PMT treatment, the RhoA activation by CNFy increased ERK phosphorylation.

Next, we asked whether PMT-induced MAPK activation is involved in the inhibition of osteoblast differentiation. The MEK-1 inhibitor PD98059 effectively abrogated the PMT effect on osteoblastogenesis in ST-2 cells and primary osteoblasts as measured by ALP activity. PD98059 also blocked PMT-induced ERK phosphorylation (Fig. 6B/C). The MAPK/ERK pathway depends on the small GTPase Ras, which is the major switch between MAPK cascade and the growth hormone receptor. We addressed the question, whether the PMT-dependent MAPK activation depends on Ras. To this end, we utilized TpeL (toxin *perfringens* large), a toxin produced by *Clostridium perfringens*. TpeL mono-O-GlcNAcylates specifically Ras and inhibits thereby Ras signaling and Ras-Raf interaction [41]. However, at higher concentrations TpeL is also known to affect Rac1. Therefore, we confirmed that in ST-2 cells and at utilized concentrations TpeL only acts on Ras but not on Rac1 (Protocol S1 and Figure S1). For further experiments a Ras-selective concentration of TpeL was used. Preincubation of ST-2 cells with TpeL inhibited PMT- and CNFy-induced ERK phosphorylation, demonstrating that MAPK activation by PMT and CNFy depends on functional Ras (Fig. 6A).

This result prompted us to study, whether the activation of MAPK depends directly on PMT-induced RhoA activity. Y27632, a known inhibitor of the RhoA-regulated Rock I and II, impaired ERK phosphorylation in ST-2 cells and primary osteoblasts (Fig. 6C). Additionally, the Gα_q/11_- induced MAPK activation was studied by using the inhibitor YM-254890 and the p63RhoGEF knockdown cells. YM-254890 abolished PMT-induced ERK phosphorylation. Most interestingly, also the knockdown of p63RhoGEF decreased PMT-induced MAPK signaling in stromal ST-2 cells (Fig. 6D). Both results implicate a Gα_q/11_ dependent transactivation of the MAPK cascade via the p63RhoGEF-RhoA-Rock axis. This signaling pathway negatively controls osteoblastogenesis.

## Discussion

Atrophic rhinitis is characterized by increased bone resorption by osteoclasts and a lack of bone regeneration by osteoblasts [8], [42]. In this study we focused on the effect of the causative agent of atrophic rhinitis, PMT, on osteoblast differentiation and activity. Osteoblasts develop from mesenchymal stem cells [43]. These stem cells are multipotent cells, having the potential to differentiate amongst others to adipocytes, osteoblasts or chondrocytes [44]. Therefore, we utilized stromal ST-2 cells derived from murine bone marrow as a cell culture model [45]. Like primary mesenchymal stem cells, ST-2 cells give rise to different cell lineages like adipocytes [46], osteoblasts [47], or hematopoiesis supporting cells [48]. Under osteogenic conditions ST-2 cells differentiate into osteoblasts. This can be determined by the measurement of various specific markers of osteoblasts like alkaline phosphatase activity. We found that PMT is able to inhibit osteogenic differentiation of stromal cells as measured by the activity and mRNA expression of the alkaline phosphatase. This effect completely depended on the catalytic action of PMT as the inactive mutant (PMT^C1165S^) exhibited no inhibitory effect on osteoblastic differentiation of ST-2 cells. The high potency of PMT is reflected by the low concentrations sufficient for impairment of cell differentiation.

Because of the high potency and effectiveness of PMT to block osteogenic differentiation, we were prompted to test, whether the toxin inhibits any kind of differentiation in stromal ST-2 cells; e.g. osteogenic and/or adipogenic differentiation. Therefore, we cultured stromal ST-2 in a medium forcing adipogenesis. Interestingly, PMT was not able to reduce differentiation of the adipocyte lineage. In line with these findings, the toxin was not able to reduce the mRNA levels of PPARγ and C/EBPα, which typically increase during adipogenesis of ST-2 cells. However, PMT even increased Oil Red O staining. Whether the observed rise in cell number completely accounts for increased Oil Red O staining or the increased expression of transcription factors like C/EBPα is additionally involved, should be clarified in further studies. If the toxin even enhances adipocyte development should also be addressed in a subsequent work.

The data indicate that PMT is not a general inhibitor of any type of differentiation but a specific inhibitor of osteoblastic but not adipogenic differentiation in stromal cells like ST-2. In another cell culture model PMT inhibited adipogenesis [49]. However, a different cell type (NIH3T3-L1) was used, which is hardly comparable with ST-2 cells.

Primary osteoblasts from newborn rat calvariae were used to verify the results obtained with the cell culture model ST-2. Martineau-Doizè and colleagues previously demonstrated that PMT-challenged rats develop atrophic rhinitis like symptoms [50]. Therefore, our model system of primary osteoblasts from rat calvariae should provide insights into the pathogenesis under veterinarian conditions. As observed for ST-2 cell differentiation, PMT strongly impaired the osteogenic development of primary osteoblasts as measured by early markers (ALP) or late markers of osteoblastogenesis (mineralization nodules). Osteoblast development is under the control of specific transcription factors. For example RUNX2 and osterix (SP7) are well known regulators of osteogenic differentiation, RUNX2 as an early transcription factor and SP7 as a late one [44]. PMT induced a significant down regulation of the transcription factor SP7, indicating that not only expression of osteoblast specific proteins, like ALP, is down regulated, but also master regulators of osteoblast differentiation are affected by PMT. However, PMT affected SP7 much stronger as compared to RUNX2. This might be due to the differentiation status of primary osteoblasts, which already undergo differentiation. Therefore, the late transcription factor SP7 might be more active and can be more efficiently regulated by PMT.

PMT reportedly affects primary osteoblasts and osteoclasts in various *in vitro* systems. Mulan and coworkers performed studies with co-cultures of osteoblasts and osteoclasts. They discussed the question whether both osteoblasts and osteoclasts are directly affected by PMT or whether the toxin targets one cell type to induce effects in the other cell type in a paracrine manner [51]. To unambiguously clarify that PMT directly acts on Gα proteins in osteoblasts, we utilized a monoclonal antibody, which selectively detects a PMT-induced deamidation [32]. This toxin-induced modification of Gα subunits was observed in osteoblastic cells indicating a direct action of PMT.

Although PMT did not impair adipocytic differentiation of ST-2 cells, we could also verify the toxin activity under adipocytic conditions. Therefore, we conclude that PMT specifically inhibits the osteoblastic differentiation of stromal ST-2 cells but not adipocytic differentiation, although G proteins are deamidated under both conditions.

To analyze the signal transduction pathway of PMT-induced blockade of osteoblastogenesis, the role of Rho proteins was studied. RhoA is a common effector of Gα_12/13_ and Gα_q/11_ and PMT was shown to stimulate RhoA via both G protein families [17], [52]. Because previous studies gave evidence for an important role of RhoA in PMT-induced blockade of osteoblast differentiation [12], we started to investigate the pathway utilized by PMT to induce RhoA activation. We observed an increased RhoA activity, after treatment of ST-2 cells and primary osteoblasts with PMT. Interestingly, a specific inhibitor of Gα_q/11_, YM-254890 [37], inhibited toxin-induced RhoA activation. These results indicate that in the cells studied, RhoA stimulation is predominantly dependent on Gα_q/11_ but not on Gα_12/13_.

In a next step we studied the effect of Gα_q/11_ inhibition on PMT-dependent blockade of osteoblast differentiation. Gα_q/11_ inhibition by YM-254890 abolished the inhibitory effect of PMT on the expression of osteoblast markers (e.g., alkaline phosphatase) during osteoblast differentiation. These results confirmed the important role of Gα_q/11_ in PMT-induced effects. Moreover, our results are in line with the studies of Ogata *et al.* showing that ectopical expression of a constitutive active mutant of Gα_q_ impairs differentiation and induces osteopenia [27], [53].

In addition, inhibition of Rock abrogated the effects of PMT on osteoblast differentiation in stromal ST-2 cells and primary osteoblasts reconfirming the pivotal role of active RhoA/Rock as a negative regulator of osteoblastic differentiation [12], [34].

Because RhoA and Gα_q/11_ play pivotal roles in PMT-induced osteoblast impairment, we were encouraged to elucidate the missing link between these signaling factors. Heterotrimeric G proteins activate RhoA via RhoGEF proteins, e.g. p115RhoGEF (Gα_12/13_) or LARG (Gα_12/13_, Gα_q/11_) [35], [54], [55]. Moreover, p63RhoGEF specifically couples Gα_q/11_ but not Gα_12/13_, to RhoA activation [36]. By immunoblot analysis we detected expression of several splice variants of p63RhoGEF in stromal ST-2 cells and primary osteoblasts. To clarify the involvement of p63RhoGEF in PMT-induced inhibition of osteoblastogenesis, we utilized an adenoviral shRNA knockdown of p63RhoGEF. p63RhoGEF knockdown in osteoblasts dramatically diminished PMT-induced RhoA activity. These findings indicate that a major portion of RhoA activation depends on the Gα_q/11_-p63RhoGEF axis. Therefore the Gαα_12/13_-induced RhoA activation via other RhoGEFs may represent only a minor part of entire RhoA activity. Finally, the inhibitory effect of PMT on osteoblastogenesis in primary osteoblasts was compared to p63RhoGEF knockdown cells. Primary osteoblasts depleted for p63RhoGEF were not affected by PMT, whereas differentiation of control cells was inhibited by PMT intoxication. These results strongly suggest that Gα_q/11_ activity is inhibitory to osteoblast differentiation. Furthermore, RhoA stimulation due to the Gα_q/11_-specific p63RhoGEF is sufficient for this effect.

The contribution of MAPK signaling in osteoblast differentiation is under discussion [40]. Inhibition of MAPK cascade at the level of growth factor receptor, MEK-1 or ERK leads to an increased differentiation of osteoblasts in different models as primary calvaria-derived osteoblasts or in pre-osteoblastic cell lines [28], [29]. It is known that PMT induces mitogenic signaling via MAPK-pathway in various cell lines as rat fibroblasts or HEK293 cells [13], [21]. Therefore, we analyzed the effect of PMT on the MAPK pathway in osteoblastic cells by measuring ERK phosphorylation. PMT was found to be a strong activator of MAPK signaling in ST-2 cells and primary osteoblasts. In line with our observation, it is described that MAPK activation blocks osteoblast differentiation. Congruently, inhibition of PMT-induced ERK phosphorylation abrogated the toxin's effect on osteoblastogenesis. Utilizing various approaches, we examined the PMT-utilized pathway to stimulate MAPK signaling in ST-2 cells and primary osteoblasts. Inhibition of Gα_q/11_, Rock and knockdown of p63RhoGEF blocked the toxin-induced ERK phosphorylation. This indicates, that PMT-activated Gα_q/11_ leads via p63RhoGEF, RhoA and Rock to a transactivation of the MAPK cascade. Inhibition of Ras by TpeL toxin, which inactivates Ras by GlcNAcetylation [41], supported the hypothesis that the transactivation of the MAPK cascade by PMT depends on functional Ras. Moreover, direct activation of RhoA by CNFy was sufficient to transduce MAPK activation in a Ras dependent manner. However, it is loosely understood how Rho/Rock signaling leads to Ras-dependent MAPK activation. It was suggested that actin dynamics provide a link between Rock and Ras activity [56].

Previously, RhoA-induced MAPK signaling has been implicated in osteoblastogenesis [40]. More recently, a genome wide analysis revealed the GEF Trio responsible for sustained MAPK pathway activation [57]. Trio contains a primary Rac-specific and a secondary Rho-specific GEF domain, of which the latter one is highly homologous to p63RhoGEF and can be activated by Gα_q/11_ [58]. Here, in osteoblastic cells, we identified p63RhoGEF as a G_q_ effector, involved in MAPK transactivation via RhoA. p63RhoGEF exhibits a restricted tissue distribution [38] and may represent a specific RhoGEF in osteoblastic cells, important for Gα_q/11_ dependent signaling. Thus, we suggest that PMT affects pre-/osteoblasts by activating the Gα_q/11_-p63RhoGEF-RhoA axis. This leads to transactivation of the MAPK pathway resulting in inhibition of the osteoblastogenesis.

Besides Gα_q/11_ PMT activates α-subunits of the Gα_12/13_ and Gα_i_ family [16]. Also these G proteins are associated with proliferative signaling, i.e. MAPK pathway stimulation [59], [60]. However, in the tested osteoblastic cells, stromal ST-2 cells and rat calvaria-derived primary osteoblasts the PMT-activated Gα_q/11_ pathway apparently prevails.

The pivotal clinical symptom of atrophic rhinitis is the atrophy of nasal turbinate bones. Over the last decades it was discovered that infections with *P. multocida* and/or *Bordetella bronchiseptica* give rise to atrophic rhinitis [61]. The virulence factors of *B. bronchseptica* and *P. multocida* are dermonecrotic toxin (DNT) and PMT, respectively. DNT activates small GTPases of the Rho family like RhoA, Rac and Cdc42 by deamidation or polyamination and impairs osteoblastogenesis [61], [62]. Our results with the specific RhoA activator CNFy strengthen the hypothesis that DNT-induced activation of the small GTPase RhoA is the key for the observed effects on bone cells. However, combined effects of DNT, directly acting on Rho GTPases, and PMT, activating Rho GTPases via Gα_q/11_-p63RhoGEF, might account for the exacerbation of the disease in the case of coinfection. A single infection with *B. bronchiseptica* causes only moderate bone loss whereas coinfection with *P. multocida* induces more drastic effects called progressive atrophic rhinitis [61]. Moreover, de Jong and Nielson recognized *P. multocida* as the causative agent of progressive atrophic rhinitis without any coinfection necessary [4]. This drastic degradation of bone tissue might be explained by the synergy of the PMT-induced effects on bone cells. On the one hand, PMT specifically inhibits osteoblastic differentiation and function and, therefore, hinders new bone formation. For this purpose, PMT utilizes a distinct signaling pathway, which we present in this work. On the other hand, PMT stimulates osteoclast activity and induces thereby a reduction of bone mass [61]. Whether the effect of PMT on osteoclast activity and/or differentiation is direct or indirect is under discussion [51]. For example, osteoclast differentiation is regulated by osteoblast-derived factors. E.g. receptor activator of NF-κB ligand (RANKL) is a positive osteoblast-derived factor for osteoclastogenesis; whereas osteoprotegerin (OPG) is a negative regulator [63]. Therefore, PMT should at least indirectly affect osteoclast development by targeting osteoblasts. In further studies it would be of interest to clarify the effect of PMT on osteoclasts and osteocytes, which also participate in bone tissue renewal.

Besides this obvious impact on bone tissue, the inhibition of osteoblastogenesis by PMT might result in a further pathogenetic advantage for *P. multocida* as a strong functional interaction of bone and the immune system takes place [64]. Recently, the involvement of osteoblasts in B cell differentiation was demonstrated [65], [66]. Additionally, it is known that PMT is a poor immunogene. Pigs suffering from atrophic rhinitis do not develop protective or specific immune response [67], [68]. Moreover, colonization of piglets with toxigenic but not with non-toxigenic strains of *P. multocida* reduces serum IgA and IgG response to ovalbumin [69], [70]. Whether the inhibition of osteoblast differentiation via the Gα_q/11_-p63RhoGEF axis is associated with these previously described immune modulatory effects of PMT should be analyzed in further studies. This would present an important function of PMT in addition to the manifest destruction of bone tissue.

**Figure 7:**
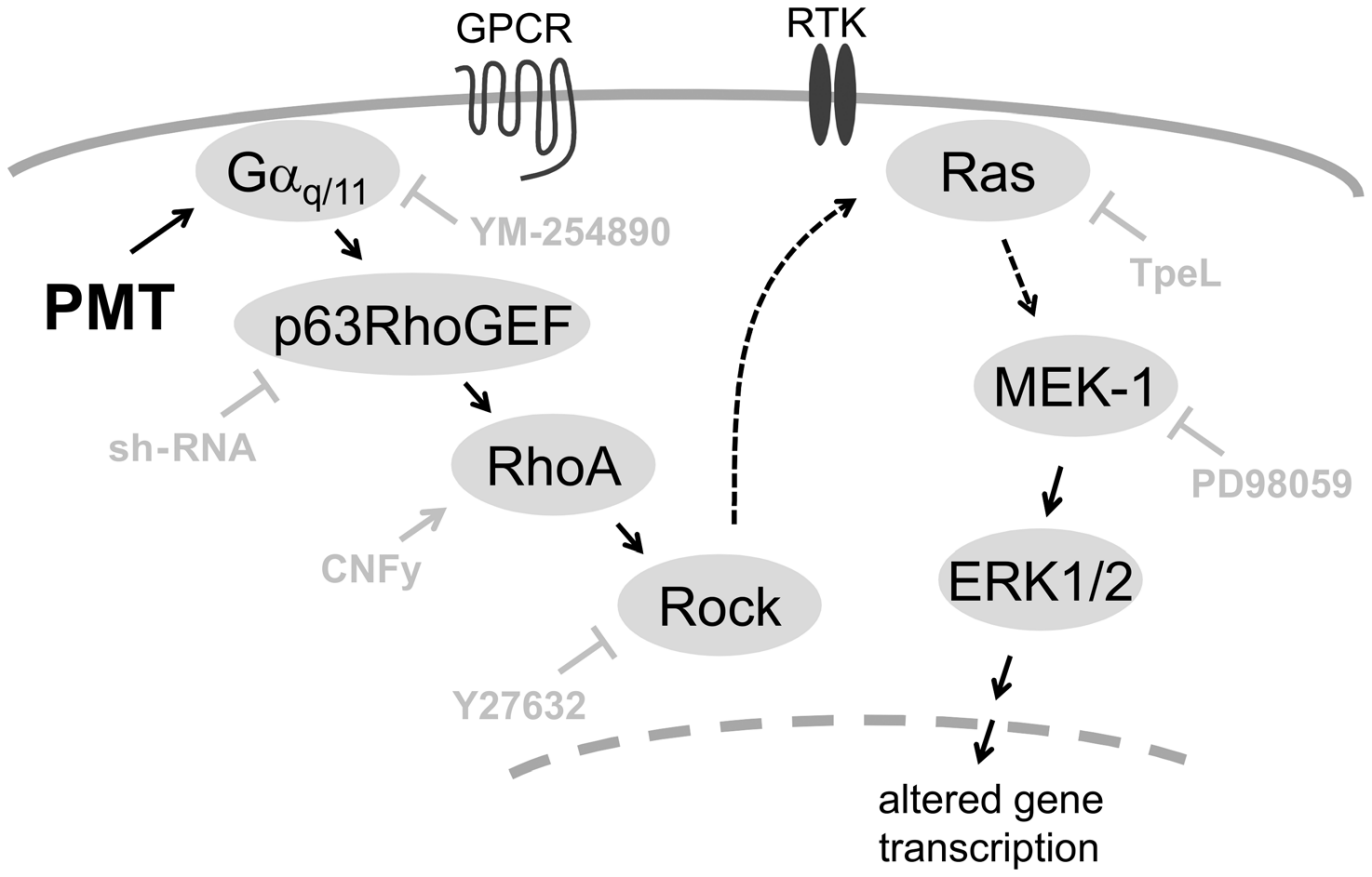
Scheme of PMT-stimulated pathway to impair osteoblastogenesis. Model of signaling pathways downstream of PMT-activated Gα_q/11_, inhibiting differentiation of osteoblasts. PMT permanently activates members of the Gα_q/11_ family. This activation is independent of any GPCR interaction. In osteoblasts, Gα_q/11_ stimulates RhoA via p63RhoGEF. RhoA-Rock stimulates in turn the MAPK pathway in a Ras dependent manner leading to altered gene expression. GPCR – G protein-coupled receptor; Rock – Rho kinase, ERK – extracellular signal-regulated kinase; RTK – receptor tyrosine kinase.

In summary, our findings indicate that PMT-induced Gα_q/11_ activation impairs osteoblastogenesis via a RhoA – MAPK pathway (Fig. 7). In pre-/osteoblasts, Gα_q/11_ and RhoA/Rock is linked by p63RhoGEF. PMT-induced RhoA/Rock activation leads to a Ras-dependent transactivation of the MAPK cascade, which is responsible for inhibition of osteoblastogenesis. Thus, the bacterial toxin PMT regulates the osteoblastic cell fate in a heterotrimeric G protein dependent manner.

## Materials and Methods

### Reagents

PCR primers were from Apara (Denzlingen, Germany). All other reagents were of analytical grade and purchased from commercial sources.

### Cell culture and lysates

Murine stromal ST-2 cells were obtained from the Leibniz Institute DSMZ (German Collection of Microorganisms and Cell Cultures) and cultivated in RPMI 1640 supplemented with 10% FCS. ST-2 cells were incubated to differentiate into adipocytes or osteoblasts in the corresponding induction medium for 10 days. The adipocyte differentiation medium (adm) consisted of RPMI 1640 (Gibco) supplemented with 10 µg/mL insulin (Sigma) and 40 ng/mL dexamethasone (Sigma). The osteoblast differentiation medium (odm) consisted of RPMI 1640 (Gibco) supplemented with ascorbic acid (284 µM) and β-glycerophosphate (200 µM). Medium including supplements was changed every two days.

Primary osteoblasts were isolated from 2–3 day old rats using collagenase digestion [71]. The crushed skulls were incubated at 37°C in RPMI 1640 Medium containing 0.1% collagenase P for 15 min per digestion cycle. Extracted cells were harvested and resuspended in RPMI 1640 Medium containing 10% FCS. Fractions 2 to 5 were pooled and used for further experiments. After 3 days cultures were trypsinized and seeded for further experiments.

Cell lysates were prepared as follows: Cells were grown to confluency and serum starved overnight. After 30 min preincubation with the indicated inhibitors the cells were incubated with PMT (1 nM, 5 h). Thereafter cells were lysed in RIPA buffer (50 mM Hepes, pH 7.4, 150 mM NaCl, 5 mM MgCl_2_, 1 mM EDTA, 1% Nonidet P-40, 0.5% (w/v) deoxycholate and 0.1% (w/v) SDS), containing complete protease inhibitor (Roche) and phosphatase inhibitor cocktail 2 and 3 (Sigma). Protein concentrations of the lysates were determined by Bradford measurement. Lysates with equalized amounts of protein were used for immunoblot analysis.

### PMT expression

PMT^wt^ and the catalytically inactive mutant PMT^C1165S^ were expressed and purified as described previously [72].

### Alkaline phosphatase activity assay

To perform the alkaline phosphatase assay cells were cultured for 10 (ST-2) or 4 (primary osteoblasts) days in odm. To measure alkaline phosphatase activity the cells were washed with PBS and then incubated with ALP assay solution (8 mM p-nitrophenylphosphate-6 H_2_O (Sigma), 12 mM MgCl_2_, 0.1 mM ZnCl_2_ and 100 mM glycine-NaOH, pH 10.5) for 10 min at 37°C. The reaction was stopped by the addition of 200 mM NaOH. The absorption was determined at 405 nm.

### Cell staining

Alkaline phosphatase staining: On coverslips cultivated cells were fixed in 4% PFA for 30 min. To remove the PFA cells were washed twice with PBS. To stain the alkaline phosphatase the cells were incubated with ALP assay solution containing 5% (v/v) ELF97 (Life Techn.) in the absence of light for 15 min at 25°C. Again cells were washed twice with PBS and incubated for 1 h with a 0.02% SYTO Green/PBS solution. Cells were mounted with Mowiol 4–88 (Carl-Roth).

van Kossa staining: Cells were stained as described by Mukherjee *et. al* 2008 [73]. In brief, cells were fixed, washed and incubated with a 5% silver nitrate solution for 30 min. After washing the cells with water the microscopic pictures were obtained.

Oil Red O staining: To detect lipid droplets, cells were stained with Oil Red O. Therefore, the cells were fixed in PFA and incubated with a saturated Oil Red O solution. Unbound Oil Red O was washed out with 70% (v/v) EtOH. To quantify the amount of Oil Red O bound to lipid droplets, the dye was extracted with a 4% (v/v) Nonidet P-40/isopropanol solution. The absorption was measured at 520 nm.

### RNA extraction and qPCR

Total RNA was extracted from either ST-2 cells or primary osteoblasts with the RNeasy Mini Kit (Qiagen). cDNA was prepared using the QuantiTect Reverse Transcription Kit (Qiagen). All Kits were used following the manufacturer's manual. Quantitative PCR was performed using GoTaq qPCR Master Mix (Promega). The expression levels of the ribosomal Protein S29 (mouse) or HPRT (rat) were used as an internal control and fold changes were calculated using the ΔΔCt method. Values are shown as 2^−ΔΔCt^.

The following primer pairs were used for analysis: mS29: forward-ATGGGTCACCAGCAGCTCTA, reverse-AGCCTATGTCCTTCGCGTACT, mALP: forward-AATGAGGTCACATCCATCCTG, reverse-CACCCGAGTGGTAGTCACAA, mPPARγ : forward-AAGACAACGGACAAATCACCA, reverse-GGGGGTGATATGTTTGAACTTG, mC/EBPα: forward-AAACAACGCAACGTGGAGA, reverse-GCGGTCATTGTCACTGGTC, rHPRT: forward-GACCGGTTCTGTCATGTCG, reverse-ACCTGGTTCATCATCACTAATCAC, rALP: forward-GCACAACATCAAGGACATCG, reverse-TCAGTTCTGTTCTTGGGGTACAT, rRUNX2: forward-CCACAGAGCTATTAAAGTGACAGTG, reverse-AACAAACTAGGTTTAGAGTCATCAAGC, rSP7: forward-CGTCCTCTCTGCTTGAGGAA, reverse-TGGAGCCACCAAACTTGC

### Immunoblot analysis

For immunoblot analysis proteins were subjected to SDS-polyacrylamide gel electrophoresis and transferred onto polyvinylidene difluoride-membrane. Anti RhoA-antibody (sc-418 (26C4)) was purchased from Santa CruzBiotech (Heidelberg, Germany), anti p63RhoGEF-antibody (51004) from Proteintech, anti pERK-antibody (4370S) from New England Biolabs, anti tubulin-antibody (T9026) from Sigma Aldrich and anti p84-antibody (ab487) from Abcam. Deamidation specific antibody anti-Gαq Q209E (3G3) was kindly provided by Dr. Y. Horiguchi (Osaka University, Japan) [32]. Enhanced chemiluminescent detection reagent (100 mM Tris-HCl, pH 8.0, 1 mM luminol (Fluka), 0.2 mM p-coumaric acid, 3 mM H_2_O_2_) was used to detect binding of the second horseradish peroxidase-coupled antibody with the imaging system LAS-3000 (Fujifilm). Quantifications of immunoblots were done using MultiGauge software.

### Rhotekin pulldown

To detect the levels of activated RhoA a Rhotekin pulldown assay was performed as described previously [17]. In brief, cells were lysed after 4 h of treatment with indicated compounds. Rhotekin-coupled beads were incubated with the lysates for 1 h at 4°C. The amount of bound and therefore active RhoA was analyzed by immunoblot analysis.

### shRNA knockdown of p63RhoGEF

For adenoviral infection cells were seeded and directly supplemented with sh-virus suspension and 8 µg/mL polybrene. A specific p63RhoGEF shRNA was used and as control a specific GFP shRNA [39]. One day after infection cells were starved for 24 h in fresh medium containing sh-virus. Cells were cultured in 10% FCS for two more days for a maximal knockdown efficacy. On day four after viral infection of the cells the described assays were performed.

### Statistics

Results are presented as means ± S.E. Significance was assessed by paired Student's t test. p values<0.05 were considered statistically significant (* = p<0.05; ** = p<0.01; ns, not significant). Multiple group comparisons were analyzed by ANOVA followed by Student's t test.

### Ethics statement

All animal experiments were performed in compliance with the German animal protection law (TierSchG). The animals were housed and handled in accordance with good animal practice as defined by FELASA (www.felasa.eu/guidelines.php) and the national animal welfare body GV-SOLAS (www.gv-solas.de). The animal welfare committees of the universities of Freiburg as well as the local authorities (Regierungspräsidium Freiburg, license X-09/31S) approved all animal experiments.

## Supporting Information

Protocol S1 **Supplemental Material and Method.**
(PDF)

Figure S1 **Inactivation of Rac1 by TpeL.** ST-2 cells were treated with Ras inactivating *Clostridium perfringens* toxin TpeL at indicated concentrations overnight. Thereafter, cells were lysed and subjected to immunoblot analysis. Rac1 was detected with glucosylation-sensitive antibody (non-glucosylated Rac1) or with glucosylation-insensitive antibody (total Rac1). TpeL modifies Rac1 only at concentrations higher than 5 nM. The Rac1 modifying *Clostridium difficile* toxin A (TcdA) was used as control (1 nM). Shown is a representative immunoblot. Quantification was done by using MultiGauge and demonstrated as fold induction normalized to untreated cells. The indicated Results are given as mean ± S.E. (n as indicated).
(PDF)
