# Supplementary material for: Pasteurella Multocida Toxin Prevents Osteoblast Differentiation by Transactivation of the MAP-Kinase Cascade via the Gαq/11 - p63RhoGEF - RhoA Axis
Source: PLoS Pathog. 2013 May 16;9(5):e1003385. doi: 10.1371/journal.ppat.1003385 (PMC3656108; doi:10.1371/journal.ppat.1003385)
Supplement: Protocol S1 — Supplemental Material and Method. (PDF) [file ppat.1003385.s001.pdf]

Protocol S1

Supplemental Material and Method

Immunoblot of Rac1

Cells were lysed in RIPA buffer. Lysates with equalized amounts of protein were used for immunoblot analysis. Rac1 was either detected with the glucosylation-sensitive antibody Mab 102 (BD Biosciences, Heidelberg, Germany) or with the glucosylation-insensitive antibody Mab 23A8 (Millipore, Eschborn, Germany).
