# Supplementary figures and images for: Pasteurella Multocida Toxin Prevents Osteoblast Differentiation by Transactivation of the MAP-Kinase Cascade via the Gαq/11 - p63RhoGEF - RhoA Axis
Source: PLoS Pathog. 2013 May 16;9(5):e1003385. doi: 10.1371/journal.ppat.1003385 (PMC3656108; doi:10.1371/journal.ppat.1003385)

Figure S1

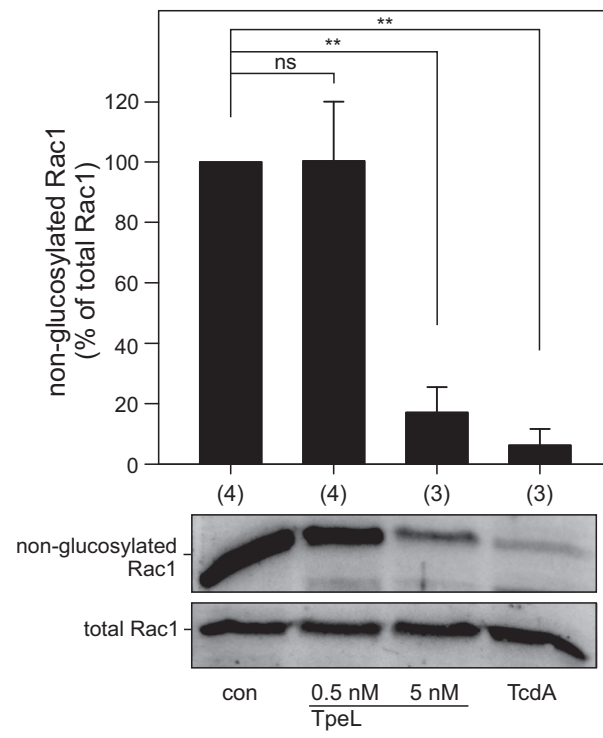

Supplement: Figure S1 — Inactivation of Rac1 by TpeL. ST-2 cells were treated with Ras inactivating Clostridium perfringens toxin TpeL at indicated concentrations overnight. Thereafter, cells were lysed and subjected to immunoblot analysis. Rac1 was detected with glucosylation-sensitive antibody (non-glucosylated Rac1) or with glucosylation-insensitive antibody (total Rac1). TpeL modifies Rac1 only at concentrations higher than 5 nM. The Rac1 modifying Clostridium difficile toxin A (TcdA) was used as control (1 nM). Shown is a representative immunoblot. Quantification was done by using MultiGauge and demonstrated as fold induction normalized to untreated cells. The indicated Results are given as mean ± S.E. (n as indicated). (PDF) [file ppat.1003385.s002.pdf]
